# Supplementary material for: Identification and functional analysis of non-coding regulatory small RNA FenSr3 in Bacillus amyloliquefaciens LPB-18
Source: PeerJ. 2023 May 15;11:e15236. doi: 10.7717/peerj.15236 (PMC10194069; doi:10.7717/peerj.15236)
Supplement: Supplemental Information 4 [file peerj-11-15236-s004.zip › KO/CK-vs-T1_map/map05230.html]

KEGG PATHWAY: Central carbon metabolism in cancer - Reference pathway


|  |  |
| --- | --- |
| **Central carbon metabolism in cancer - Reference pathway** |  |

[
Pathway menu
| Organism menu
| Pathway entry
| Show description
| User data mapping
]

Malignant transformation of cells requires specific adaptations of cellular metabolism to support growth and survival. In the early twentieth century, Otto Warburg established that there are fundamental differences in the central metabolic pathways operating in malignant tissue. He showed that cancer cells consume a large amount of glucose, maintain high rate of glycolysis and convert a majority of glucose into lactic acid even under normal oxygen concentrations (Warburg's Effects). More recently, it has been recognized that the 'Warburg effect' encompasses a similarly increased utilization of glutamine. From the intermediate molecules provided by enhanced glycolysis and glutaminolysis, cancer cells synthesize most of the macromolecules required for the duplication of their biomass and genome. These cancer-specific alterations represent a major consequence of genetic mutations and the ensuing changes of signalling pathways in cancer cells. Three transcription factors, c-MYC, HIF-1 and p53, are key regulators and coordinate regulation of cancer metabolism in different ways, and many other oncogenes and tumor suppressor genes cluster along the signaling pathways that regulate c-MYC, HIF-1 and p53.

|  |  |  |
| --- | --- | --- |
| Reference pathway | 184% 150% 122% 100% 82% 67% 55% | 图片下载 |
